# Exploring a cost-effective and straightforward mechanism for uninterrupted *in situ* maximum wave runup measurements

**DOI:** 10.1098/rsta.2024.0182

**Published:** 2024-09-09

**Authors:** Deborah Villarroel-Lamb, Richard R. Simons

**Affiliations:** ^1^ Department of Civil and Environmental Engineering, Faculty of Engineering, The University of the West Indies (UWI), St Augustine Campus, Trinidad and Tobago; ^2^ Department of Civil, Environmental and Geomatic Engineering, Faculty of Engineering Sciences, University College London (UCL), London, UK

**Keywords:** maximum wave runup, in-bed water level, porous bed, static bed, moveable bed, Newton International fellowship

## Abstract

Wave runup, the excess water level above mean sea level, has been measured using different techniques with varying degrees of precision and associated practical limitations. This critical parameter, typically included in coastal assessment studies, varies temporally and spatially and depends on variables that include beach characteristics and nearshore hydrodynamics. Access to continuous datasets, using efficient mechanisms can assist resource-limited regions, such as Caribbean small-island developing states (SIDS), in overcoming coastal resilience obstacles. Experiments were conducted at University College London (UCL) and the University of the West Indies (UWI), which were designed to explore the temporal behaviour of the water surface within the bed during runup events. The experiments encompassed linear waves impacting a static porous bed (UCL) and a moveable granular beach (UWI), with pressure sensors buried at the base of each beach. The analyses showed that the averaged values of the time-varying water elevations within the bed, when spatially presented, produced a quadratic or cubic polynomial fit, where the curves’ stationary points were accurate indicators of the location of the maximum runup position at the surface of the bed. In this way, an arrangement of buried pressure sensors can be used as an efficient means to accurately produce a continuous time series of maximum runup positions.

This article is part of the theme issue ‘Celebrating the 15th anniversary of the Royal Society Newton International Fellowship’.

## 1. Background and introduction

Swash zone hydrodynamics and swash sediment transport remain areas of ongoing investigation owing to their importance in identifying critical coastal zone planning and management elements such as shoreline response or effects on groundwater [1–4]. In particular, as coastal areas face multiple threats including those from natural and anthropogenic hazardous events, which may be exacerbated by uncertain climate changes, it is essential that there is an improved understanding of the physical characteristics of this important coastal zone [4–6]. The swash zone is that part of the nearshore area between the maximum run up limit on the landward side, and the minimum rundown limit on the seaward side. The exact extent of the zone varies with water level changes, such as those caused by tides and storm surges, as well as the characteristics of the beach face, nearshore bed conditions and hydrodynamics. There have been numerous studies that investigate various aspects of the swash zone; numerical modelling, physical modelling and field data campaigns are common methods of data collection used to improve the knowledge and understanding of the physical processes in this complex zone.

Many researchers have investigated wave runup and presented parametric formulations to predict runup magnitude, which usually include offshore wave conditions, a suitable beach slope and bed features [2,7]. The wave runup is the combination of two quantities that vary temporally and spatially: one is time-varying swash, the other is wave set-up, which is a time-averaged quantity [4,8,9]. Wave runup is a random variable and is typically described probabilistically; two common measurements used to define extreme runup limits are the maximum wave runup, ***R*_max_**, and the runup exceeded by 2% of incoming waves, ***R_u2%_*** [4,9,10]. There are different methods used to capture runup data, including point instruments such as pressure sensors or ultrasonic sensors, photogrammetry, LiDAR, traditional surveying techniques, resistance wire gauges (or runup wires) and video camera images. Each method of data collection has varying degrees of accuracy, practical limitations, differences in the extent and frequency of data collected and different methods of analyses for data extraction [11]. Traditional surveying techniques may be used for measuring wave runup and involve the collection of data through visual observations which can be subjective, and total-station surveying techniques where the data can be geo-referenced and reduced to mean sea level [12]. However, traditional surveying techniques, which require personnel present on site during data acquisition, do not allow for a continuous mode of data collection. Dual resistance wires or runup wires can be placed across the beach face and have been used by various researchers [4,8,13]. However, phase errors can occur at higher frequencies with runup wires, and the apparatus can be easily disturbed by external factors such as interference from beachgoers and animals [4,8,11]. Additionally, there can be difficulty in obtaining accurate measurements for a thin swash lens, and maximum runup may be underestimated in these cases [14]. Video cameras have become quite common for mapping the swash front; they can be cost-efficient and allow for runup estimates even during high-energy events [4,11]. However, video camera data processing can be laborious and time-consuming; there is also difficulty in tracking swash oscillations and a requirement to have adequate ground-truthing data for verification. In addition, the resolution, height and distance of the camera from the swash zone can produce errors, especially when attempting to discern the runup extent [14]. Furthermore, continuous data collection can be hampered during low visibility conditions (e.g. rain and fog during the day, and nighttime conditions) [15]. Terrestrially based LiDAR systems can be costly, and requires infrastructure that prohibits rapid deployment. There are also errors that may result from the use of LiDAR such as inaccuracies in the surface elevation from reflections or unspecified movement of the scanner [16]. Laser scanners require the use of threshold values because of the noise generated in the runup measurements and they may produce values that are larger when compared to visual measurements [17]. In one study, the laser scanner reflections were used as a proxy optical measurement and produced errors ranging between 0.8 and 8.6% (with an average of 4%) when compared with visual measurements [17]. Low-cost LiDAR solutions are being investigated to offer cost-effective options for continuous time series of water-level changes. The results of a pilot study with a prototype low-cost LiDAR produced a root-mean-square difference of 0.039 m (rising tide) and 0.032 m (falling tide) in water elevation when compared with a modern terrestrial LiDAR [18]. Ultrasonic sensors produce measurements similar to two-dimensional laser scanners, but with a slightly lower error [19]. However, the accuracy of the measurements depends on the number of sensors used and instrument set-up may be laborious. Pressure sensors have been used in various studies and also provide a cost-effective alternative to measuring wave runup on beaches. There are various methods for extracting the maximum runup values from the pressure sensor records, with differing levels of computational complexity [4,16,19,20]. Moreover, pressure sensors have been used in a variety of field conditions, from rocky slopes to sandy beaches, but the changes on non-cohesive sediment beaches may pose some challenges [4]. Researchers sometimes implement a combination of wave runup measurement techniques to improve the accuracy of the output, as was the case with the use of a laser scanner and video techniques to obtain a root mean square error (RMSE) ranging from 1.7 to 3.2 cm in laboratory experiments [19].

Other researchers have sought to focus on the hydrodynamics of this zone including both super-surface and sub-surface patterns [3,21–23]. One area of research that is relevant to sub-surface flows in the swash zone is in determining how the flow patterns through the porous beach media affect the swash dynamics and hence sediment movement. In the swash, water infiltration and exfiltration patterns have been deemed significant to beach morphology [24–26]. There have been multiple investigations into the sediment transport regime and resulting beach changes as this zone experiences significant sediment movement during erosion and accretion events [27–30]. However, the complexity of the swash zone limits a one-size-fits-all approach for multiple hydrodynamic forcings and beach characteristics. This is readily illustrated in the application of wave runup parameterizations at various coastal sites. An assessment of various commonly used runup formulae found that the RMSE can range from 0.48 to 1.20 m in field cases with correlations ranging from 0.31 to a maximum of 0.62 [7]. Nonetheless, research has still sought to deduce patterns and trends from existing data in a bid to provide broad solutions to this challenging problem.

Many coastal areas, particularly small-island developing states (SIDS), are especially vulnerable to coastal hazards (e.g. hurricanes and tsunamis) and this vulnerability is expected to be exacerbated by the effects of climate change [30,31]. Caribbean SIDS have coastal regions that are diverse and which exhibit differing coastal characteristics. There are different levels of exposure to hazards, varying types of biogeography and beaches. Beaches have varying features; mildly sloping and sandy, steep-sloped (even near vertical) cliff, rocky shorelines or wetland areas [32]. Coastal hazards such as erosion and flooding are present, as well as other future concerns regardless of the type of shoreline [31,32]. Emphasizing the fact that even within the regional setting, the vast diversity of coastal features can make it difficult to achieve a single solution to a problem across Caribbean SIDS. Given that many SIDS have access to limited resources, a viable solution to data collection is of paramount importance to underpin decisions and to achieve coastal resilience in these communities. It is, therefore, imperative to explore effective, easily-implemented and cost-efficient options for Caribbean coastal regions to produce comprehensive and accurate measurements of key coastal parameters [18].

This investigation, which started at University College London (UCL) in 2015, sought to investigate the specific relationship between changes in water level within the bed and the maximum runup position. One specific intention was to identify a non-intrusive, cost-effective means to readily and accurately determine the maximum wave runup, in a continuous manner, without using remote observations such as video cameras which required time-consuming post-collection analyses, or LiDAR which is cost-prohibitive. Using buried pressure sensors, simple post-collection methods can yield a continuous time series of runup maxima regardless of visibility and which are unaffected by external interference. The UCL experiments were done using a fixed, plane, porous beach and were completed in November 2015. The results were promising, and it was decided to assess the suitability of the approach using a moveable granular bed, which is the case in many field conditions. Therefore, a series of similar experiments, using a mobile bed, were conducted between September 2019 and February 2020 at the University of the West Indies (UWI). We first present the experimental and analytical methods executed at both UCL and UWI in §2. Then, the results are presented in §3 and discussed in §4, with the conclusions presented in §5. The results of this study demonstrate a practical means to obtain wave runup measurements using low-cost equipment and fast processing methods to yield reasonably accurate results that can be used for effective decision-making in Caribbean SIDS.

## 2. Methods

There are two main attributes to this investigation: the physical experiments and the subsequent data post-processing. In the sub-sections below, the methods used at UCL and at UWI are discussed; although similar there are differences in both the physical modelling and the ensuing analyses.

### (a) Methods at UCL

#### (i) Experimental methods

**Figure 1.**
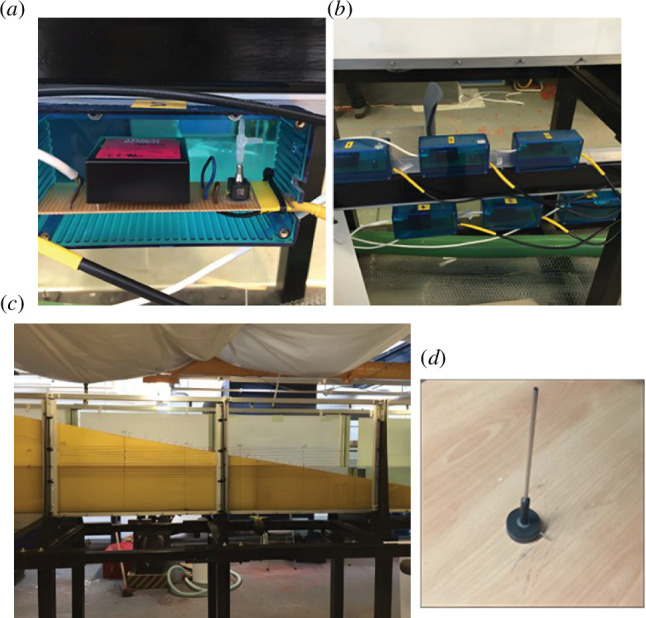
Various components for the UCL experiments: (*a*) Data translation board set-up showing connected pressure sensor; (*b*) all six pressure sensors; (*c*) artificial foam beach; (*d*) inserts into foam beach to connect to pressure sensor.

**Figure 2.**
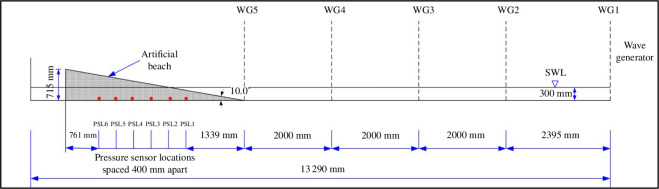
Sketch of the UCL experimental set-up of wave gauges (WG1–WG5) and pressure sensors located at the base of the artificial beach at pressure sensor locations PSL1–PSL6.

The experiments were conducted in a wave-current flume, which is located in the Department of Civil, Environmental and Geomatic Engineering at UCL. The flume is approximately 13.4 m long × 0.7 m deep × 0.45 m wide with working water depths in the range 0.2 to 0.5 m. A wave generator with active wave absorption, capable of producing regular and random waves, was positioned at one end while an artificial beach, with a planar beach slope of 10°, made of engineered foam, was at the other end. Different artificial beaches of varying porosities were obtained using different types of foam, but the beach slope remained unchanged. Specific R30, R45 and R80 foams were used with laboratory-tested permeabilities of 0.401, 0.105 and 0.041 m s^−1^, respectively. The permeability of each of the foams was determined using a constant head permeameter. Six Honeywell sensing and control pressure sensors (50 mmHg 40PC001B1A), placed 400 mm apart, were used to measure the in-bed pressures of the artificial beach; the first sensor was located 1.339 m from the toe of the beach. A variety of investigations were conducted during the assessment phase of the experimental set-up for the pressure sensors; however, this article reports on those tests where hollow metal tubes were used to specify the location of the pressure measurements within the foam. The top of the hollow metal tubes (4.27 mm internal diameter and 0.25 mm wall thickness) were located 240 mm above the base of the flume and were connected to the pressure sensors using high-strength silicone tubing (internal diameter of 2 mm and a wall thickness of 0.5 mm). The elevation was chosen to ensure that the openings remained submerged for the selected waves and for the working water depth of 300 mm. When the wave tank was filled, the pressure head in the tank allowed the silicone tubing to be readily filled as these exited the base of the tank. Each pressure sensor was powered by Tracopower transformers which supplied the required 5 V to each sensor (see figure 1). The data translation board (DT 9800-BNC) connected the pressure sensors to a computer where the QuickDAQ 2014 software was used to display the outputs. The sensors were calibrated daily by increasing and decreasing the water depth in the flume using eight different elevations: two readings at the still water level (SWL); 10, 20, 30 and 50 mm above the SWL; and 10, 20 and 30 mm below the SWL. At each elevation during calibration, 15 s of data were collected. Five resistance wave gauges were located along the length of the wave flume; figure 2 illustrates the wave-gauge and pressure sensor placement. Each wave gauge was calibrated daily prior to the start of the experiment by moving the wave gauges vertically at each location to the same elevation changes as those used for the pressure sensor calibration; data were collected for 15 s. The wave gauges were connected to the same data translation board as the pressure sensors and the QuickDAQ 2014 software was again used to record these data.

Data were extracted as text files containing 11 output channels (five for the wave gauges and six for the pressure sensors). For the experiments, a total of 25 linear wave conditions were used and consisted of all the possible combinations of five wave amplitudes: 0.02, 0.03, 0.04, 0.05 and 0.06 m and five wave frequencies: 0.3, 0.35, 0.5, 0.7 and 1.0 Hz. Data were collected for the pressure sensors and the wave gauges every 0.015 s (approximately 66.667 Hz) for a total of 90 s. A measurement grid was drawn on the external glass wall of the wave tank in the location of the swash zone. The grid was used to make direct visual observations of the maximum wave runup position for each wave condition and beach type using data from a video camera mounted at the side of the flume. This is a common method used in laboratories for accurate runup measurements [4]. The maximum wave runup position was measured and recorded both as a vertical elevation above the toe of the beach and as a horizontal distance from the beach toe. This vertical elevation could be easily converted to the commonly used runup magnitude (which is the vertical elevation in excess of the SWL) by subtracting the SWL elevation above the toe; however, the maximum wave runup, ***R*_max_**, given as a vertical elevation above the toe of the beach is used in this article.

#### (ii) Analytical methods

First, using the calibration files of the pressure sensors and wave gauges, the averaged values obtained over the 15 s data sample facilitated the conversion of the voltage readings to water elevations in metres. It was expected that wave reflection would be an issue for some conditions in the flume, so the wave reflection coefficients were determined using the Lin and Huang approach [33]. A histogram was plotted for the wave runup data using the Freedman–Diaconis rule to select the bin widths. To ensure suitability of the output, only the last 60 s of recorded data were used for both the pressure sensors and the wave gauges for any subsequent analyses. One of the pressure sensors, the one originally located at PSL5 (pressure sensor location 5—see figure 2), failed during the experiment and the sensor originally located at PSL6 was moved to PSL5. As a result, only five of the original six sensors were operational for the full duration of the experiments and only relevant output channel data from PSL1 to PSL5 were used for the analyses. The water levels varied temporally at each pressure sensor location. For each experimental run, the time-varying water-level statistics from each pressure sensor were extracted from the data. These statistics included the maximum, minimum and averaged water levels at each sensor location over the specified 60 s recorded period. The distance from the pressure sensor location to the toe of the artificial beach was used as the independent variable, ***x***, and each water-level statistic was used, in turn, as the dependent variable (***y*_max_**, ***y*_min_** and ***y*_mean_** for the maximum, minimum and mean values, respectively), which was dependent only on ***x***. The best fit (using a least-squares approach) quadratic (second degree) and cubic (third degree) polynomial for each experimental run were generated and the coefficients retained (see equations (2.1) and (2.2)). A straight line fit was not appropriate as this relies upon the inherent assumption that the water level continues to increase with increasing distance from the beach toe, which is not physically reasonable. Higher-order polynomials were avoided owing to the number of available pressure sensors, as well as to ensure the resulting polynomial remained physically appropriate and the post-processing was straightforward. Therefore, there is one quadratic and one cubic polynomial for each experimental run and for each water-level statistical value used. Subsequently, the coefficient of determination (***R^2^***) value was used as a statistical measure of the correlation between the observed values of ***y*_max_**, ***y*_min_** and ***y*_mean_**, and the predicted values using the fitted polynomial. Next, the derivative of each polynomial was determined to ascertain stationary points, that is, local maxima, minima or inflection points. At stationary points, the derivative is zero and the roots of the derivative equation were obtained to identify the ***x*** value associated with these stationary points. There is one root for the derivative equation of the initially fitted quadratic polynomial, and two roots for the derivative equation of the initially fitted cubic polynomial. It was anticipated that the roots of the derivative equation would represent the horizontal distance from the beach toe to the maximum runup location and hence give an indication on the beach face of the position of the maximum runup. With this expectation, the real component of each polynomial derivative root, ***x_R_***, was then used to calculate ***R*_max_** given as a vertical elevation from the toe of the beach using the slope and basic trigonometry. The percentage error between the ***R*_max_** derived from this analysis and the directly observed ***R*_max_** was calculated relative to the observed values. These errors were computed for all experimental runs and the overall averages, as well as the averages for each bed permeability, were determined. The percentage error is the relative difference between the wave runup values obtained using the method with the pressure sensors, ***X_P_***, when compared with the visual measurement, ***X_V_***, expressed as a percentage, as shown in equation (2.3). In addition, the relationships between the experimental parameters of wave reflection, wave characteristics, bed permeability, polynomial coefficients and percentage errors were explored using correlation analyses where the correlation coefficient, ***R***, was used. Linear regression was used in this study to readily explore and assess the relationships between the various parameters:


 (2.1) $$y_{quadratic}=p_{1}x^{2}+p_{2}x+p_{3},$$

(2.2) $$y_{cubic}=p_{1}x^{3}+p_{2}x^{2}+p_{3}x+p_{4},$$

(2.3) $$\%\;error=\frac{X_{V}-X_{P}}{X_{V}}\;\times \;100.$$

### (b) Methods at UWI

#### (i) Experimental methods

The experiments were conducted in a wave flume approximately 10 m long × 1.0 m deep × 0.55 m wide with working water depths between 0.3 and 0.7 m. Similar to the UCL experiments, the wave generator at one end of the wave tank had active wave absorption and was capable of producing regular and random waves. An artificial composite beach was situated at the other end, which comprised blocks of loosely cemented coarse-to-medium well-sorted gravel at the lower portion of the beach. All cavities in the artificial beach and the upper portion of the beach, comprised loose medium well-sorted gravel particles (of average size 9.53 mm; tested permeability of 0.3231 m s^−1^); the composite beach had a final plane slope of 35°. Gravel particles in this topmost mobile layer of the bed were free to move during experiments.

**Figure 3.**
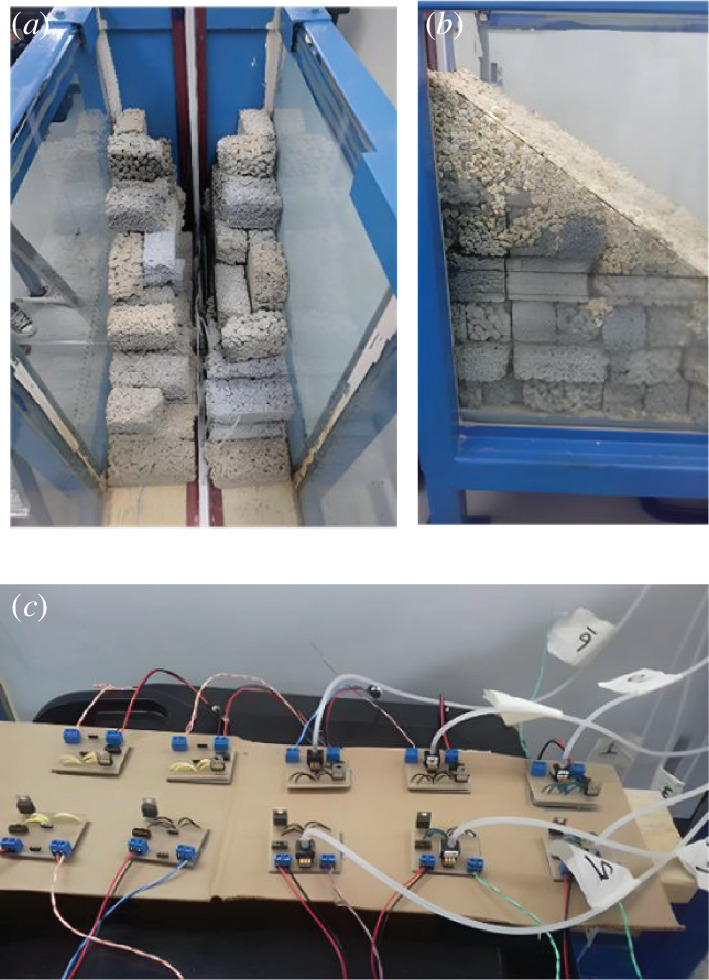
Various components for the UWI experiments: (*a*) Layout of plastic straws inside the artificial beach; (*b*) completed artificial beach and (*c*) circuit board set-up for pressure sensors.

**Figure 4.**
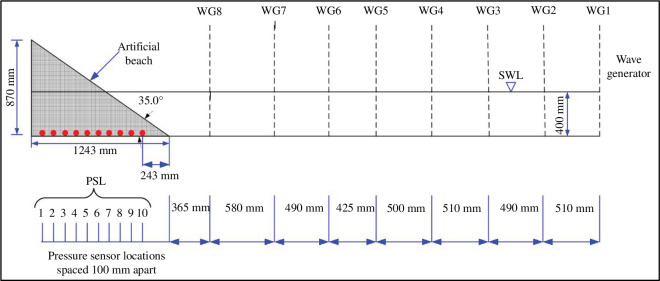
Sketch of the UWI experimental set-up of wave gauges (WG1–WG8) and pressure sensors located at base of the artificial beach at pressure sensor locations PSL1–PSL10.

Under the artificial beach, an enclosed PVC plastic conduit was fastened centrally to the base of the wave tank and secured to 10 lengths of flexible clear silicone tubing (each 2 mm internal diameter and 4 mm external diameter; tubes were of varied lengths). Within the bed, the silicone tubing exited the top of the PVC plastic conduit through 10 equidistant 7 mm diameter openings spaced 10 cm apart; the first opening was 10 cm from the back wall of the wave tank. Each length of the flexible silicone tubing was secured inside long, rigid, plastic hollow cylinders (i.e. straws, each approx. 279.4 mm long), the ends of which did not protrude from the top of the cylinders. As at UCL, the rigid straws were used to ensure that the submerged end of the flexible tubing maintained a fixed position during the experiment and that they remain submerged for the working 400 mm water depth for the selected wave conditions. The other end of each length of the flexible silicone tubing exited the wave tank at the top of the back wall. At the start of the experiment, the wave tank was filled to the desired working water depth, i.e. 400 mm. Water was syphoned through each length of flexible tubing and care was taken to avoid trapped air within the tubing. The unsubmerged ends of the flexible tubing were attached to 10 Honeywell pressure sensors (50 mmHg 40PC001B1A) mounted on separate circuit boards. In this arrangement, eight pressure sensors were connected to submerged openings that were located within the artificial bed, and the two remaining sensors were connected to submerged openings protruding from the artificial beach, but seaward of the SWL (see figure 3). Each circuit board was fitted with a switch, battery terminals to connect 9 V batteries, terminals for the National Instruments USB-6009 8-Input−14bit Multifunction I/O data logger and a 5 V regulator to step down the 9 V battery voltage to the required 5 V for the pressure sensors. The data logger has a maximum of eight inputs; the eight pressure sensors located at PSL1 to PSL8 (see figure 4) that were connected to flexible tube openings within the artificial bed were used during the experiment to measure the water level variations within the bed. Sensor PSL1 was closer to the rear of the artificial beach and PSL8 was closer to the toe (0.44 m from the toe). Sensors PSL1 to PSL8 were used to collect data for all experimental runs while the remaining two pressure sensors, lPSL9 and PSL10, were used to independently measure the water levels seaward of the SWL on the artificial beach. Openings connected to sensors PSL9 and PSL10 were not buried in the bed but were only connected for one wave condition (i.e. UWI_WC20: 0.04 m amplitude and 1.1 Hz frequency) replacing sensors PSL2 and PSL1 respectively, on the data logger. The pressure sensor sampling rate for data collection was 1000 Hz and the Laboratory Virtual Instrument Engineering Workbench (LabVIEW) Software was used to retrieve the data and extract .lvm files, which are text-based measurement files from LabVIEW. Each day, prior to the start of data collection, all pressure sensors were calibrated using five different water-level positions: at a working water depth of 400 mm and 9 and 18 mm above and below this water depth. During calibration, pressure sensor data were collected for a period between 65 and 115 s at each water-level position. Timing of the data collection duration was not well-controlled for the pressure sensors, and exact timing was not achieved. In addition to the pressure sensors, eight wave gauges (WG1–WG8) were located along the length of the wave flume. Gauge WG1 was closest to the wave generator and WG8 was closest to the artificial beach (at a distance 0.365 m from the toe). Figure 4 illustrates the wave-gauge placement and pressure sensor locations. Each wave gauge was calibrated daily prior to the start of the experiment by moving the wave gauges vertically through five different levels: at the working water depth of 400 mm and at 50 mm and at 100 mm below and above this water depth. During calibration, the proprietary software, Njord, provided by the wave generator manufacturer, Edinburgh Designs, was used, allowing all wave-gauge output to be given in metres.

A total of 20 linear wave conditions comprising all the possible combinations of four wave amplitudes: 0.01, 0.02, 0.03 and 0.04 m, and five wave frequencies: 0.7, 0.8, 0.9, 1.0 and 1.1 Hz, were used. This resulted in six wave conditions that were similar to the UCL experiments. Each wave condition was run three consecutive times to account for any variability in the results; water levels were allowed to settle between runs. During the experimental runs, waves were generated for a total of 70 s, but only 60 s of wave data were collected from the wave gauges. Data were collected from the pressure sensors for a total of approximately 70 s and the beach was re-profiled after each experimental run. A transparent measurement grid was fixed to the external glass wall of the wave tank in the location of the swash zone. This grid, which was zeroed at the water level of the working water depth, was used to make direct visual observations of the ***R_max_*** position for each experimental run, again, complemented by images from a camera mounted at the side of the flume. The maximum vertical extent of the water above the SWL for each run was marked and then the distance was measured using the grid. Similar to the UCL experiments, the ***R_max_*** value was recorded as a vertical elevation and a horizontal distance from the toe of the beach.

#### (ii) Analytical methods

Using the pressure sensor calibration files, the averaged values for the last 55 000 data points were used to convert the voltage data to elevations in metres. There was a reset in the pressure sensors every 1000 readings resulting in missed readings and the 55 000 data points did not translate to exactly 55 s of data. This posed no issue during calibration as the averaged values were calculated. For all subsequent analyses, only the last 55 000 data points of recorded data were used from the pressure sensor data to maintain consistency of the output. Other than these stated differences, the analytical methods were similar to those executed at UCL to derive estimates of the location of ***R_max_*** given as a vertical elevation from the toe of the beach. The errors between the ***R_max_*** calculated in this way and the observed values were also derived in a similar manner to the methods used at UCL.

The water-level data, for the UWI_WC20 run, from the two unburied pressure sensors PSL9 and PS10, along with the remaining buried sensors PSL3, PSL4, PSL5, PSL6, PSL7 and PSL8, served to independently validate the water levels within the artificial beach. First, to generate a smooth curve near the zero line, a sum-of-sines model with five terms was fitted to the water-level readings from WG8 and the data from the sensors PSL3 to PSL10. This was done for each run and was used to determine the location of all zero crossings. The zero crossings were then used to estimate the mean zero crossing wave height, ***mH_z_***, and the mean zero crossing wave period, ***mT_z_***, at WG8 and at PSL3 to PSL10. Wave gaugeWG8 was located in the full working water depth of 0.4 m. However, it should be noted that the buried sensors PSL7 and PSL8, and the unburied sensors PSL9 and PSL10 recorded water levels on the beach face at locations below (or seaward of) the SWL in reduced water depths of approximately 0.02, 0.09, 0.16 and 0.23 m, respectively, with PSL7 very close to the intersection of the SWL with the beach face. The pressure sensors at locations PSL3–PSL6 recorded data on the beach but at locations landward of the SWL.

## 3. Results

The results from UCL and UWI are presented separately in §§3.1 and 3.2, respectively.

### (a) Results from UCL experiments

**Figure 5.**
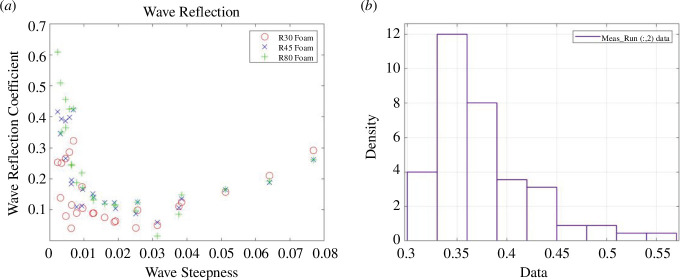
UCL experimental data; (*a*) Reflection coefficients plotted against the wave steepness parameter and (*b*) histogram showing the distribution of the observed maximum wave runup as a vertical elevation in m from the toe of the beach.

In figure 5*a*, the reflection coefficients are plotted against a dimensionless wave steepness parameter (***H/L_o_***) where the wave height, ***H***, is twice the wave amplitude and ***L_o_*** is the deep-water wavelength derived from linear wave theory. The wave reflection data are listed in electronic supplementary material, table S49, and the water-level statistics at each pressure sensor location are listed in electronic supplementary material, tables S1–S3.

**Figure 6.**
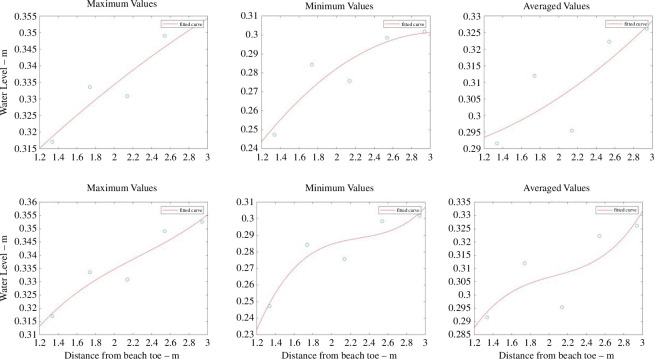
Samples of the quadratic and cubic polynomial fit of the maximum, minimum and mean (average) water levels (*y*-axis) to the distance from beach toe (*x*-axis) using the UCL data; (*a*) to (*c*) are the quadratic fits and (*d*) to (*f*) are the cubic fits.

**Figure 7.**
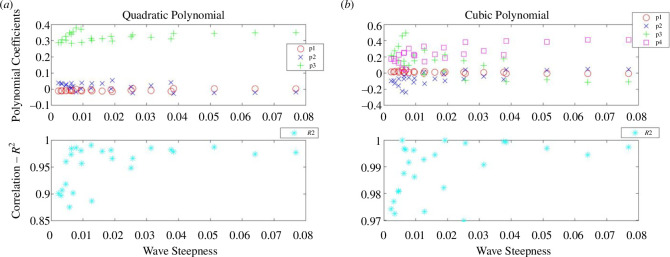
*R*^2^-correlation and the coefficients of the fitted polynomials (*p_1_, p_2_, p_3_ and p_4_*) using the maximum water levels at each sensor plotted against the wave steepness parameter for the R30 foam beach; (*a*) the quadratic polynomial and (*b*) the cubic polynomial.

**Figure 8.**
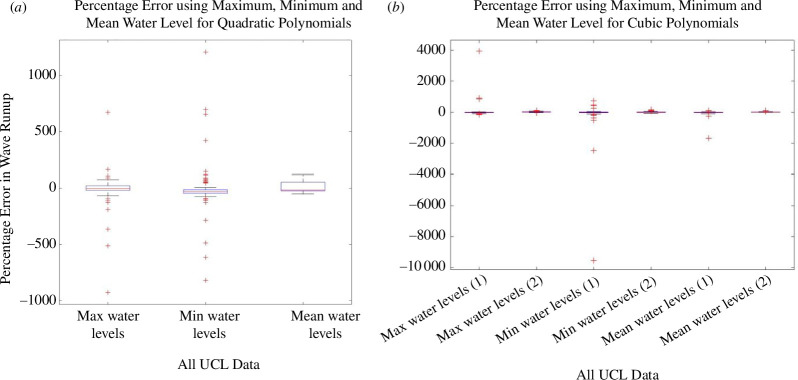
Box and Whisker plots of the percentage error in calculated wave runup for all the UCL data using the maximum, minimum and mean water levels at the pressure sensors; (*a*) uadratic polynomial fits; and (*b*) cubic polynomial fits.

**Figure 9.**
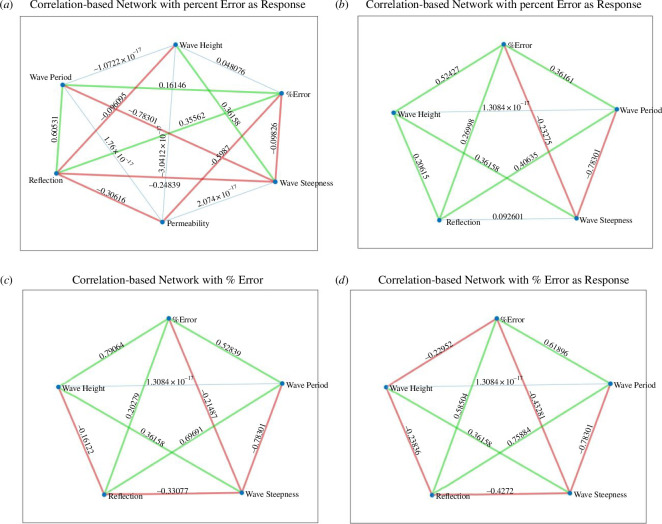
Correlation (*R*) - based network plots for UCL data using the quadratic polynomial fit and the percentage error in calculated wave runup as the response variable; (*a*) All the available data; (*b*) data for R30 foam only; (*c*) data for R45 foam only and (*d*) data for R80 foam only.

**Figure 10.**
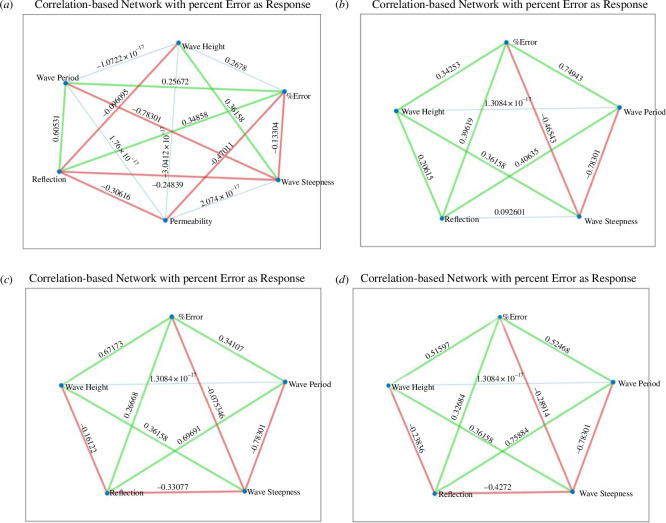
Correlation (*R)* - based network plots for UCL data using the cubic polynomial fit and the percentage error in calculated wave runup as the response variable; (*a*) All the available data; (*b*) data for R30 foam only; (*c*) data for R45 foam only and (*d*) data for R80 foam only.

**Figure 11.**
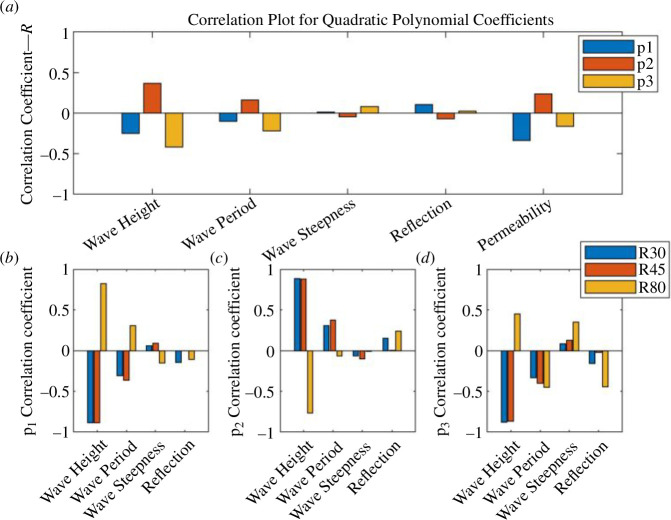
Plots showing the correlation (*R*) for the UCL data using the quadratic polynomial fit between each polynomial coefficient (*p_1_, p_2_ and p_3_*) and other selected experimental variables; (*a*) all the available data; (*b*)–(*d*) show the differences in correlation for the R30, R45 and R80 foam data.

**Figure 12.**
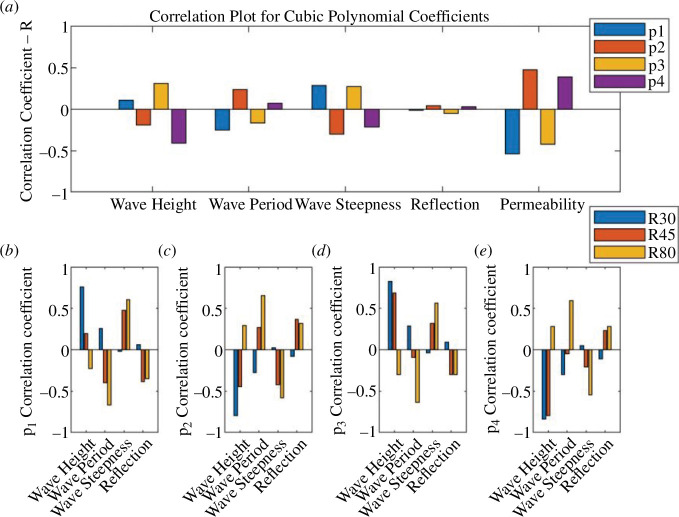
Plots showing the correlation (*R*) for UCL data using the cubic polynomial fit between each polynomial coefficient (*p_1_*, *p_2_*, *p_3_* and *p_4_*) and other selected experimental variables; (*a*) all the available data; (*b*)–(*e*) show the differences in correlation for the R30, R45 and R80 foam data.

The directly observed wave runup information is listed in electronic supplementary material, tables S7–S9, and the histogram showing this observed runup distribution is given in figure 5*b*. Sample plots are provided in figure 6 to illustrate the polynomial fits of the given water level statistic from each sensor location with respect to its distance from the toe. The coefficients of the quadratic and cubic fits are listed in electronic supplementary material, tables S13–S21. A sample of the data extracted for the polynomial fits is provided in figure 7 where the polynomial coefficients and respective correlations are plotted against ***H/L_o_***. The percentage errors between the calculated and the directly observed vertical elevation of ***R*_max_** from the beach toe are listed in electronic supplementary material, tables S31–S39, and illustrated in electronic supplementary material, figures S1–S8. Figure 8 provides a representation of the results for all the data. A summary of the percentage errors is listed in table 1 where values are averaged over the specified dataset. The correlations between the various experimental parameters including the percentage error are shown in figures 9 and 10; figures 11 and 12 show the correlation between the experimental parameters and the polynomial coefficients.

**Table 1.** Summary of the averaged values of the percentage error in calculated wave runup and the correlation coefficient for the UCL data where values are averaged over the specified dataset.

data set	all data	all data	R30 foam	R30 foam	R45 foam	R45 foam	R80 foam	R80 foam
**curve fitting**	**quadratic**	**cubic**	**quadratic**	**cubic**	**quadratic**	**cubic**	**quadratic**	**cubic**
**water-level statistic**	**maximum**	**maximum**	**maximum**	**maximum**	**maximum**	**maximum**	**maximum**	**maximum**
correlation coefficient	0.82	0.89	0.95	0.99	0.74	0.87	0.77	0.82
percentage error vertical elevation from toe to maximum runup [1]	−11.41	39.29	34.38	−56.99	−12.07	−32.84	−56.54	207.71
percentage error vertical elevation from toe to maximum runup [2]	n/a	15.84	n/a	9.83	n/a	3.55	n/a	34.14

**water-level statistic**	**minimum**	**minimum**	**minimum**	**minimum**	**minimum**	**minimum**	**minimum**	**minimum**
correlation coefficient	0.81	0.85	0.97	1.00	0.91	0.92	0.56	0.64
percentage error vertical elevation from toe to maximum runup [1]	−6.30	−163.18	−40.09	−66.36	−8.92	−427.22	30.12	4.06
percentage error vertical elevation from toe to maximum runup [2]	n/a	−4.10	n/a	−30.72	n/a	7.39	n/a	11.04

**water-level statistic**	**mean**	**mean**	**mean**	**mean**	**mean**	**mean**	**mean**	**mean**
correlation coefficient	0.79	0.86	0.89	0.98	0.94	0.99	0.53	0.60
percentage error vertical elevation from toe to maximum runup [1]	10.71	−53.42	−18.94	−60.67	−19.26	−109.05	70.34	9.46
percentage error vertical elevation from toe to maximum runup [2]	n/a	1.56	n/a	−7.10	n/a	−7.45	n/a	19.24

### (b) Results from the UWI experiments

**Figure 13.**
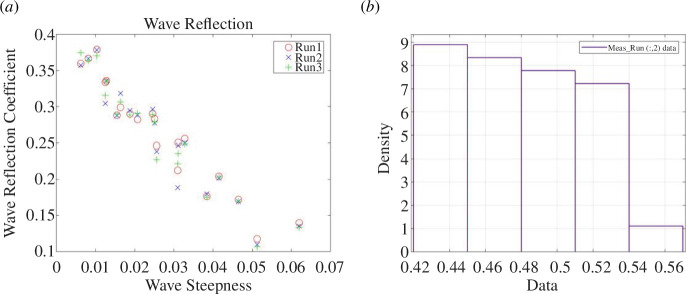
UWI Experimental data; (*a*) Reflection coefficients plotted against the wave steepness parameter; (*b*) Histogram showing the distribution of the observed maximum wave runup as a vertical elevation in m from the toe of the beach.

**Figure 14.**
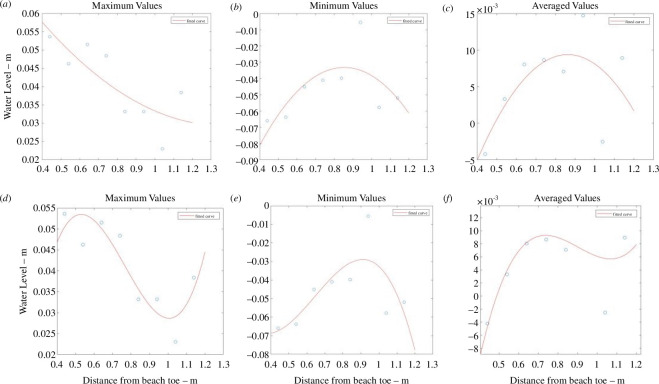
Samples of the quadratic and cubic polynomial fit of the maximum, minimum and mean (average) water levels (*y*-axis) to the distance from beach toe (*x*-axis) using the UWI data; (*a*) to (*c*) are the quadratic fits and (*d*) to (*f*) are the cubic fits.

**Figure 15.**
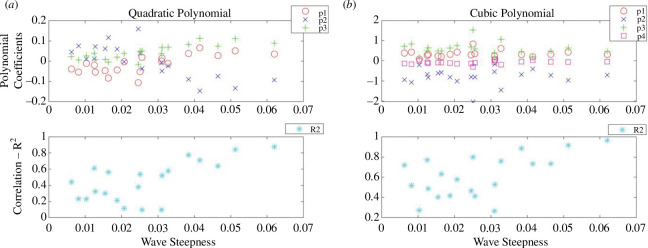
The *R*^2^-correlation and the coefficients of the fitted polynomials (*p_1_*, *p_2_*, *p_3_* and *p_4_*) using the maximum water levels at each sensor plotted against the wave steepness parameter for run 1; (*a*) the quadratic polynomial and (*b*) the cubic polynomial.

The reflection coefficients for these experiments are shown in figure 13*a* and can also be found in electronic supplementary material, table S50. The full dataset of the directly observed wave runup can be found in electronic supplementary material, tables S10–S12. The histogram showing the directly observed wave runup distribution is given in figure 13*b*. All the water level statistics at each pressure sensor location are listed in electronic supplementary material, tables S4–S6, and the coefficients of the quadratic and cubic fits are listed in electronic supplementary material, tables S22–S30. As with the UCL data, sample plots are provided in figure 14 to illustrate the polynomial fits of the given water level statistic from each sensor location with respect to its distance from the toe. A sample of the data extracted for the polynomial fits is provided in figure 15 where the polynomial coefficients and correlations are again plotted against ***H/L_o_***.

**Figure 16.**
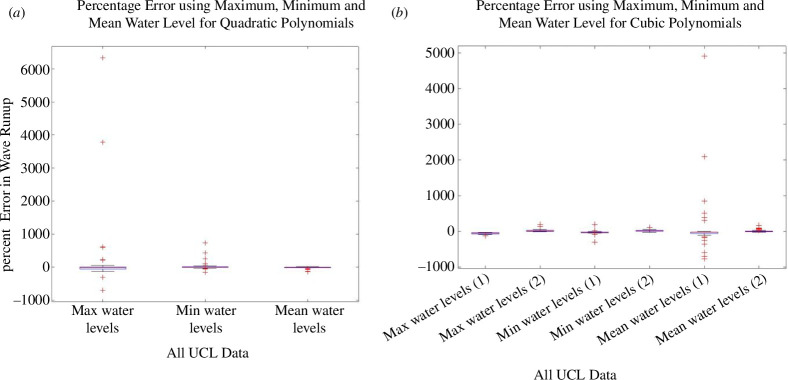
Box and Whisker lots of the percentage error in calculated wave runup for all the UWI data using the maximum, minimum and mean water levels at the pressure sensors; (*a*) quadratic polynomial fit and (*b*) cubic polynomial fit.

**Figure 17.**
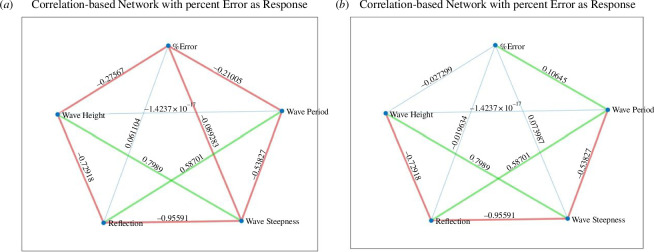
Correlation (*R*)-based network plot for run 1 of UWI data using the percentage error in calculated wave runup as the response variable; (*a*) the quadratic polynomial fit and (*b*) the cubic polynomial fit.

**Figure 18.**
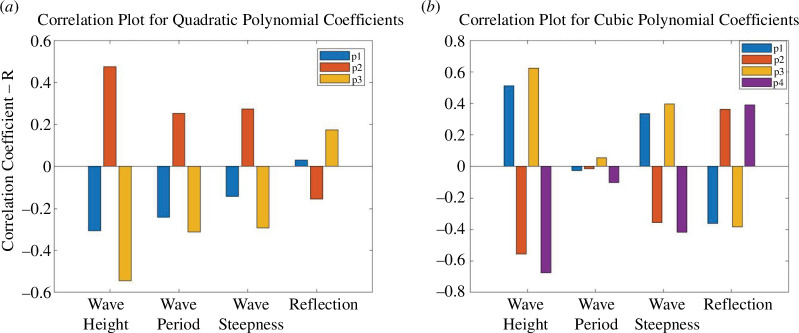
Plots showing the correlation (*R*) for run 1 of the UWI data between each polynomial coefficient and other selected experimental variables; (*a*) *p_1_*, *p_2_* and *p_3_* for the quadratic polynomial fit and (*b*) *p_1_*, *p_2_*, *p_3_* and *p_4_* for the cubic polynomial fit. (*a*) Correlation plot for quadratic polynomial coefficient and (b) Correlation plot for cubic polynomial coefficient.

The percentage errors between the calculated and the directly observed positions of ***R*_max_** are listed in electronic supplementary material, tables S40–S48, and illustrated in electronic supplementary material, figures S9–S16. Once more, for the sake of completeness, figure 16 is included which show the errors for all the data. The summary of the errors is listed in table 2, which again shows values averaged over the specified dataset. The summary of the independent validation of the pressure sensors with the data at WG8 is listed in table 3. The correlation between all the experimental parameters including the percentage error is given in figure 17; and figure 18 shows the correlation with the polynomial coefficients for Run 1 only.

**Table 2.** Summary of the averaged values of the percentage error in calculated wave runup and the correlation coefficient for the UWI data where values are averaged over the specified dataset.

data set	all data	all data	run 1	run 1	run 2	run 2	run 3	run 3
**curve fitting**	**quadratic**	**cubic**	**quadratic**	**cubic**	**quadratic**	**cubic**	**quadratic**	**cubic**
**water-level statistic**	**maximum**	**maximum**	**maximum**	**maximum**	**maximum**	**maximum**	**maximum**	**maximum**
correlation coefficient	0.46	0.61	0.45	0.61	0.44	0.58	0.48	0.64
percentage error vertical elevation from toe to maximum runup [1]	156.49	−53.71	−34.78	−53.93	−36.57	−55.90	540.83	−51.29
percentage error vertical elevation from toe to maximum runup [2]	n/a	19.81	n/a	17.09	n/a	19.32	n/a	23.02

**water-level statistic**	**minimum**	**minimum**	**minimum**	**minimum**	**minimum**	**minimum**	**minimum**	**minimum**
correlation coefficient	0.40	0.62	0.40	0.64	0.39	0.58	0.41	0.63
percentage error vertical elevation from toe to maximum runup [1]	22.38	−29.27	19.60	−18.33	12.83	−41.47	34.71	−28.01
percentage error vertical elevation from toe to maximum runup [2]	n/a	16.80	n/a	14.59	n/a	18.90	n/a	16.92

**water-level statistic**	**mean**	**mean**	**mean**	**mean**	**mean**	**mean**	**mean**	**mean**
correlation coefficient	0.43	0.47	0.43	0.46	0.44	0.47	0.43	0.46
percentage error vertical elevation from toe to maximum runup [1]	−20.22	71.12	−19.47	255.11	−20.28	75.91	−20.92	−117.67
percentage error vertical elevation from toe to maximum runup [2]	n/a	8.04	n/a	11.44	n/a	5.05	n/a	7.62

**Table 3.** Summary of the zero-crossing parameters using the water-level data from pressure sensors located at locations PSL3 to PSL10 and the water-level data from the wave gauge WG8.

	WG8	PSL3	PSL4	PSL5	PSL6	PSL7	PSL8	PSL9	PSL10
**run 1**									
**mH_z_** (m)	0.1016	n/a	n/a	0.0211	0.0224	0.0450	0.0571	0.0602	0.0643
**mT_z_** (s)	0.9092	n/a	n/a	0.8064	0.5809	0.7782	0.9100	0.9094	0.9098
**run2**									
**mH_z_** (m)	0.1023	n/a	n/a	0.0204	0.0248	0.0465	0.0623	0.0589	0.0660
**mT_z_** (s)	0.9094	n/a	n/a	0.9087	0.6726	0.8330	0.9098	0.9094	0.9098
**run 3**									
**mH_z_** (m)	0.1029	n/a	n/a	0.0201	0.0166	0.0470	0.0586	0.0579	0.0649
**mT_z_** (s)	0.9095	n/a	n/a	1.1047	0.4045	0.9087	0.9101	0.9095	0.9100

## 4. Discussion of results

The wave reflection coefficients were generally smaller in the UCL dataset than the UWI data. This was attributed to the shorter wave flume length at UWI as well as the milder beach slope at UCL, which resulted in less wave reflection as more energy is dissipated over the beach. The reflection coefficients increased with decreasing bed permeability for the UCL data, which was confirmed in figures 9 and 10; again, this is an expected result as less water infiltrates the bed and less energy is lost through this mechanism. For the UCL dataset, there was considerable variability in the magnitudes of the directly observed wave runup, which may be because of the relatively milder slope of the artificial beach which produced larger (more easily distinguishable) runup values for similar wave conditions when compared with the UWI directly observed wave runup, which showed little variability in the data. The direct measurement of the wave runup was similar for both the UCL and the UWI methods, so this was only a factor in that the visual method using the background grid does not readily allow for smaller changes in the runup position to be discerned.

The higher observed correlation in the UCL data between the fitted polynomials and the water levels was probably caused by there being fewer sensor locations and possible water-level errors caused by experimental set-up at UWI. For the UCL experiment, the silicone tubing connecting the measurement locations inside the beach exited the base of the flume and allowed for gravity to ensure no trapped air was present. However, in the UWI experiment, the flexible tubing ran up and then exited the top of the back of the flume, increasing the risk of trapped air and discontinuous flow which may have resulted in some erroneous readings at various sensor locations. The experimental set-up at UWI, however, provides a good simulation of the field environment where a similar approach will probably be adopted in future work. During the UWI experiments, much effort was made to prevent the occurrence of flow discontinuities and resulted in pressure sensors data that were qualitatively satisfactory (see table 3); that is, the sensors generally produced similar wave periods when compared with the closest wave gauge, and also exhibited expected reduced wave heights. There is an expected steady reduction in wave height magnitudes moving towards the back of the beach where the wave periods were generally the same as the expected value of 0.9091 s except at PSL6. The ‘n/a’ in table 3 refers to where no zero crossings were available to make an estimate of ***mH_z_*** and ***mT_z_***. In addition, it is not known how the homogeneity of the artificial beach material might have affected the results; the foam beach at UCL was more homogenous than the moveable bed at UWI; however, the experiments at UWI were closer to the field case where the bed is not generally homogenous.

The average percentage error for the UCL data, using a quadratic fit, was less than 12% (refer to table 1 and figure 8). Using minimum water levels produced the smallest average error but had a larger spread in the error data. Using mean water levels produced the smallest dispersion in the error, improving the precision of the result. Using the cubic polynomial fit, one root of the derivative equation, expectedly, gave a better result and was considered the most suitable solution. Generally, for the UCL data the percentage error (with the cubic fit) was less than 16% when using all the data. Using minimum or mean water levels produced the smallest average error, but the spread was marginally smaller using mean water levels. Referring to table 2 and figure 16, the average percentage error for the UWI data, using a quadratic fit, was greater than for the UCL data; using minimum or mean water levels produced the smallest average error of approximately 20%. The variability, however, was smaller for mean water levels because minimum water levels had a larger spread in the error data. For the cubic polynomial fit to all the data, the result associated with the most appropriate root of the derivative equation, had an average error of less than approximately 20%, whereas using mean water levels produced the smallest error of approximately 8%. The smallest dispersion in the error was associated with maximum water levels. Overall, using mean water levels consistently produced more precise results in locating the maximum runup position from both the quadratic and the cubic polynomial fits, although use of minimum water levels demonstrated similar predictive capability in the UCL data; to a lesser extent, maximum levels also showed some useful predictive information in the UCL data (but for the UWI data with the cubic polynomial fits only). The highest polynomial degree was three, selected at UCL, primarily based on the number of pressure sensor locations and a requirement to ensure that the resulting polynomial remained physically appropriate. The quadratic polynomial fit was judged to be best in representing the spatial distribution of the physical water levels within the bed, and from the UCL experiments produced a good outcome in predicting the position of ***R_max_***, but the results with the quadratic polynomial fit were not well reproduced in the UWI experiments. Expectedly, the mean errors were greater when compared with the study by Hofland *et al*. [17], where a comparison of wave runup measurements using a laser scanner to visual measurements was carried out. However, the method here uses lower-cost equipment and simple post-processing to provide a good estimate of the *in situ* wave runup.

While, generally, correlations were not strong for the assessment of the percentage error and polynomial coefficients of the experimental variables, there is some useful information to be gleaned. Referring to the correlation between the percentage error and the experimental parameters in the UCL data: the error was better correlated to the bed permeability, wave reflection, wave period, wave height and wave steepness. The wave height had stronger correlations with the percentage error in the cubic fit and for constant permeability beaches. For the UWI data: the percentage error in the quadratic fit to the inbed water levels was best correlated to the wave height and wave period only; bed permeability was not assessed in the UWI experiments as only one bed permeability was used. For the cubic fit to the UWI data, the percentage error appears to be best correlated to the wave period only. The differences in the parameter dependence on the percentage error may be attributed to differences in the bed slope, including improved accuracy in obtaining the directly observed wave runup from the UCL experiments. Referring to figures 7 and 15 (and electronic supplementary material, tables S13–S30), the UCL and the UWI data produced very similar polynomial coefficients across the entire dataset for both the quadratic and cubic polynomial fits, where the similarity is more marked for higher wave steepnesses, the cubic fits and in the UCL data. This consistency in the polynomial values supports the use of a polynomial to delineate the water levels within the bed. With respect to the better-correlated parameters to the polynomial coefficients (refer to figures 11, 12 and 18), there were analogous correlation trends between the polynomial coefficients and the experimental variables when compared with the percentage error; with wave steepness becoming more important for these polynomial coefficients. The increased significance of wave steepness in the polynomial coefficients is also observed in the UWI data. The correlations conclusively demonstrate that the fitted polynomials are related to the beach and wave parameters, which are typically used in wave runup prediction formulae and thus can reflect changes in wave runup location.

Some of the limitations associated with this experimental study have already been highlighted, the most significant are: only using visual measurements as the true measurement; not including random waves as part of the experimental design; not investigating the effect of bed homogeneity on the results; not assessing the effect of bed permeability on a mobile bed; not including an investigation of the impact of the depth of pressure sensor reading; not optimizing the number and locations of the pressure sensors; and not performing a true field test, where a comparison with the main wave runup empirical formulae might have been obtained. At the design stage for the UWI experiments, it was believed that the higher number of pressure sensors would yield a better result, but the close proximity of the sensors may have adversely affected the results. That is the reason why it would be useful to assess if any relationship exists between the spacing of the buried sensors and the accuracy of the prediction of the wave runup.

To obtain a continuous record of *in situ* wave runup for field or long-term deployment of a series of buried pressure sensors, the procedure is quite straightforward. First, with reference to figures 6 and 14, which show the polynomial fits, it appears to be useful to have at least one pressure sensor located landward of the expected maximum runup position to adequately locate the relevant polynomial stationary point. For quick analyses, the time-averaged values of the water levels from the pressure sensors can be fitted to a quadratic polynomial with the distance of the sensors from a reference datum serving as the dependent variable. The averaging can be done for any selected period of time e.g. 30 or 60 s segments, but must account for the fact that the bed permeability will affect the time of infiltration. Using a quadratic polynomial will yield a single result for the maximum wave runup position; even at lower accuracy this is a critical improvement in coastal assessments at data-sparse sites. A limited separate wave runup data collection campaign using high-accuracy instrumentation will then serve to validate results and adjust the wave runup time series accordingly. The use of a cubic fit improves the accuracy of determining the ***R*_max_** position, and without any other available data, the optimal root can be selected based on its proximity to the output from the quadratic fit. In this way, even with limited funding, technical capability and human resources, data-sparse regions can yield continuous *in situ* output of this critical coastal parameter.

## 5. Conclusion and recommendations

The results of these experiments illustrate definitively that a system of buried pressure sensors can be applied to estimate the location of the maximum wave runup by fitting the averaged water levels at each sensor to a polynomial. The use of a quadratic polynomial was judged satisfactory in producing reasonable estimates of the maximum wave runup position, and adopting this approach has an improved practical application as it produces only one stationary point on the curve, which can easily yield the maximum runup position. Cubic polynomials can be applied, depending on the number of pressure sensors; however, the two possible roots of the derivative require an interpretive means to discern the most appropriate root in the absence of any other measurements. Fitting water levels to higher polynomials is not recommended for this reason. In addition, pressure sensors should be buried at sufficient depth as to remain below the water level for all expected wave conditions and water-level changes. In this way, an unintrusive and continuous mechanism can be applied to measure the *in situ* maximum wave runup position for laboratory or field deployment. In the field, where silicone tubing may be long, care must be taken to ensure the tubing remains filled with water and a suction gun or pump may be used during set-up. The tubing should also remain unclogged, which can easily be achieved using a wire mesh at the mouth of the tapping. For data analyses in a continuous mode, data can be segmented into selected times and analyses executed over these periods to determine the maximum runup. It is expected that these periods should be large enough to encompass at least one wave runup and sufficiently long enough to allow for any infiltration to occur. Therefore, in less permeable beds the analysis period may be increased; it is expected that this methodology will fail in near impermeable beds. However, for countries where coastal data is scarce, and critical decisions must be made at coastal sites, this cost-effective and easily applied method of collecting *in situ* wave runup measurements will provide much-needed data to advance site assessments and in so doing improve the resilience of vulnerable coastal communities.

## Data Availability

Additional results are available via the supplementary material [34].
